# Pancreatic β-Cell Death in Response to Pro-Inflammatory Cytokines Is Distinct from Genuine Apoptosis

**DOI:** 10.1371/journal.pone.0022485

**Published:** 2011-07-29

**Authors:** J. Jason Collier, Susan J. Burke, Mary E. Eisenhauer, Danhong Lu, Renee C. Sapp, Carlie J. Frydman, Shawn R. Campagna

**Affiliations:** 1 Department of Nutrition, University of Tennessee, Knoxville, Tennessee, United States of America; 2 Department of Chemistry, University of Tennessee, Knoxville, Tennessee, United States of America; 3 Sarah W. Stedman Nutrition and Metabolism Center, Duke University Medical Center, Durham, North Carolina, United States of America; 4 University of Tennessee Obesity Research Center, Knoxville, Tennessee, United States of America; Pennington Biomedical Research Center, United States of America

## Abstract

A reduction in functional β-cell mass leads to both major forms of diabetes; pro-inflammatory cytokines, such as interleukin-1beta (IL-1β) and gamma-interferon (γ-IFN), activate signaling pathways that direct pancreatic β-cell death and dysfunction. However, the molecular mechanism of β-cell death in this context is not well understood. In this report, we tested the hypothesis that individual cellular death pathways display characteristic phenotypes that allow them to be distinguished by the precise biochemical and metabolic responses that occur during stimulus-specific initiation. Using 832/13 and INS-1E rat insulinoma cells and isolated rat islets, we provide evidence that apoptosis is unlikely to be the primary pathway underlying β-cell death in response to IL-1β+γ-IFN. This conclusion was reached via the experimental results of several different interdisciplinary strategies, which included: 1) tandem mass spectrometry to delineate the metabolic differences between IL-1β+γ-IFN exposure versus apoptotic induction by camptothecin and 2) pharmacological and molecular interference with either NF-κB activity or apoptosome formation. These approaches provided clear distinctions in cell death pathways initiated by pro-inflammatory cytokines and bona fide inducers of apoptosis. Collectively, the results reported herein demonstrate that pancreatic β-cells undergo apoptosis in response to camptothecin or staurosporine, but not pro-inflammatory cytokines.

## Introduction

Type 1 diabetes mellitus is an autoimmune disease that occurs when pancreatic β-cells within the islets of Langerhans are selectively destroyed by resident and invading immune cells [1]. The initial trigger for onset of this process is unknown. However, leukocyte secretion of pro-inflammatory cytokines, such as IL-1β and γ-IFN, is a critical factor in the destruction of islet βcells [2]. IL-1β induces the expression of several genes that produce inflammatory mediators, including inducible nitric oxide synthase (iNOS); the intracellular production and accumulation of nitric oxide impairs β-cell function and decreases viability in both rodent and human islets and β-cell lines [3], [4], [5]. While the toxic effects of pro-inflammatory cytokines have been known for decades [6], [7], [8], the mechanism underlying β-cell death in response to these cytokines is not well understood; delineating the pathway(s) of β-cell death in response to pro-inflammatory cytokines is essential for targeting precise and robust strategies for protection against losses in functional β-cell mass. Currently, β-cell death in response to IL-1β and γ-IFN has been suggested to proceed through either necrosis [9], [10], [11] or apoptosis [12], [13].

Apoptosis is a form of programmed cell death classically described by morphological changes that include rounding of cells, changes to membrane and cytoskeletal structure, and DNA fragmentation [14]. These alterations occur through the biochemical actions of a cysteine aspartate protease family of enzymes called caspases, which are usually present as inactive zymogens that require proteolytic processing in response to a particular stimulus to become active [15]. Activation of executioner caspases, such as caspase-3, results from the assembly and processing of initiator caspases (e.g., caspase-9) in response to a signal, such as DNA damage. Part of this caspase activation process relies on oligomerization of the pro-apoptotic proteins Bak and Bax, which releases cytochrome c into the cytoplasm via permeabilization of mitochondrial membranes [16]. Cytosolic cytochrome c then interacts with APAF-1 and caspase-9 to form the apoptosome, a large protein complex which also contains dATP [17]; the apoptosome processes pro-caspase-9 to active caspase-9, which then proteolytically cleaves caspase-3. Cleaved (active) caspase-3 targets a large number of intracellular substrate proteins for degradation, leading to cellular termination. Once the apoptotic cascade has been initiated, multiple cellular targets are cleaved by executioner caspases in a highly organized, ATP-dependent manner [15]. Because of the efficient nature of this pathway, apoptosis is the usual method by which metazoans replace damaged or unwanted cells during normal growth and development; consequently, this cellular death mechanism is usually associated with immune tolerance rather than initiation of immune responses [15], [18].

Alternatively, cells undergoing necrosis display losses in plasma membrane integrity, declining ATP levels, and tend to provoke inflammatory responses [18], [19]. A number of pro-inflammatory signals, including IL-1β, produce inflammation via activation of the NF-κB pathway [20], which is a dimer consisting of various combinations of five different DNA-binding subunits: p65 (RelA), RelB, c-Rel, p50 and p52.The activity of these transcription factors is regulated at multiple levels, including cytoplasmic retention by the inhibitors of kappa B (IκB) proteins [21]. IL-1β activates the interleukin receptor type I at the cell surface, leading to recruitment of receptor adaptor proteins, and signaling through various kinases, including the IκB kinases (IKKs). The IKKs phosphorylate the IκB proteins, promoting their degradation and releasing NF-κB proteins to translocate from the cytoplasm to the nucleus. Once in the nucleus, the different NF-κB subunits can hetero- and homo-dimerize to transactivate a multitude of genes (e.g., iNOS) involved in inflammatory responses that lead to tissue dysfunction and cell death [5], [22]. Release of cellular contents via this type of death mechanism can activate immune cell responses. Thus, dying cells are managed differently by the immune system depending on whether they are undergoing apoptosis or necrosis [19], [23], [24], [25].

The present study was undertaken to investigate the mechanism of β-cell death in response to pro-inflammatory cytokines. Currently, the accepted means of defining necrotic cell death is to demonstrate that apoptosis is not occurring [18]. Towards this end, we have previously demonstrated that interference with pro- and anti-apoptotic proteins impacts β-cell viability in response to true apoptotic signals, but has no effect on pro-inflammatory cytokine-mediated cell death [11]. Alternatively, other studies have suggested an activation of the intrinsic pathway of apoptosis by cytokines [13]. Therefore, to further address the mechanism underlying β-cell death in response to cytokines, we have taken the following approaches: 1) temporal- and concentration-dependent investigation of the biochemical effects of pro-inflammatory cytokines vs. bona fide inducers of apoptosis ; 2) a tandem mass spectrometry based metabolomics study to quantitate the metabolic signatures produced by the pro-inflammatory cytokines IL-1β and γ-IFN as compared to camptothecin, a bona fide apoptotic inducer; 3) pharmacological and molecular inhibition of signaling through the NF-κB pathway; and 4) manipulation of the apoptosome components APAF-1 and caspase-9. Collectively, the results reported herein confirm and extend previous observations [9], [10], [11] that apoptosis is not the major mechanism of β-cell death in response to pro-inflammatory cytokines.

## Materials and Methods

### Cell Culture, Adenoviral Vectors, Nitrite and Viability

All animal work was performed in accordance with procedures approved by the Duke University IACUC in appropriately accredited facilities (Animal Protocol # A309-10-12). Culture of the INS-1-derived 832/13[26] and INS-1E [27] cell lines have been described. Islets were isolated as described [11]. Recombinant adenoviruses expressing βGAL [28], IκBα S32/36A [29] and caspase-9 dominant-negative [30] have all been described. Recombinant adenovirus expressing a luciferase gene under the control of a multimerized NF-κB promoter was a gift from Dr. Michael Karlstad (University of Tennessee Graduate School of Medicine). Nitrite production and viability measured by both MTS and adenylate kinase release have been described previously [11].

### Caspase 3 Enzymatic Activity

Whole cell lysates were collected using radioimmunoprecipitation assay (RIPA) buffer and analyzed for caspase -3 enzyme activity using Caspase Glo substrate (Promega) and a GloMax plate reader (Promega) according to manufacturer's protocol.

### Transfection of siRNA Duplexes

832/13 cells were transfected with pre-annealed siRNA duplexes (siAPAF: siRNA ID# 52648) from Ambion/Applied Biosytems using DharmaFECT reagent 1 (Dharmacon) according to the manufacturer's protocol. Suppression of target genes was analyzed by real-time RT-PCR detection of mRNA levels and functional outcome.

### Isolation of Protein and Immunoblot Analysis

Whole cell lysates were harvested using the M-PER lysis buffer (Pierce). Protein concentration was determined via BCA assay (Pierce) using bovine serum albumin as the standard. Immunoblotting was performed as described previously [11], [31]. Antibodies were from Santa Cruz Biotechnology (IκBα), Cell Signaling (caspase-3) and Sigma (tubulin and β-Actin).

### Isolation of RNA, cDNA Synthesis and Real-time RT-PCR

Total RNA was isolated from cells using Isol-RNA Lysis Reagent (5 Prime) according to the manufacturer's protocol. Verso cDNA synthesis kit (Thermo Scientific) was used for first-strand synthesis of cDNA using 0.5 µg of RNA. For real-time PCR measurements of relative mRNA abundance, 2.5% of the total reverse transcriptase (RT) reaction was used with Taqman primer/probe sets (Applied Biosystems; siAPAF1 Cat# Rn0057683) and ABsolute Blue QPCR Master Mix (Thermo Scientific). Real-time RT-PCR was performed using the Applied Biosystems Prism 7300 sequence detection system and software. Relative mRNA levels for individual genes were normalized to either cyclophilin or ribosomal S9.

### Sample Preparation for Mass Spectrometric Analysis

832/13 cells were grown to confluence in 15 cm dishes and treated as indicated in the figure legends. At the end of the treatment period, cells were washed twice with ice cold PBS, scraped and pelleted at 500× g; the aspirated dry cell pellets were immediately flash frozen using liquid N_2_. Four individual experiments were performed in duplicate on each of 3 days. Prior to extraction, the collected cell pellets were weighed, and 30–50 mg of each was placed into a microcentrifuge tube at −78°C. A solution of cold methanol and water (8∶2 v/v, 1.5 mL) kept at −80°C was then added to the tube to extract the metabolome from the cells. The contents were mixed by vortexing and allowed to sit at the extraction temperature for 20 min. Particulates were then removed from the samples by centrifugation, and the supernatants were transferred to autosampler vials for further analysis by the LC-MS/MS methods described below.

### Chromatographic Separation Methods

High performance liquid chromatographic (HPLC) separation of the metabolites was performed via a slight modification of previously reported methods [32], [33]. Details for these techniques are as follows: A quaternary pump was used to generate a gradient for the elution of compounds from the stationary phase. In total, two HPLC runs are necessary to fully characterize the metabolome for each sample since separate detection for positively ionizing and negatively ionizing compounds is required. For all samples, 10 µL was injected onto the column via an Finnigan Surveyor Autosampler (Thermo Electron) cooled to 4°C. For positive mode analyses, a Luna 5 µ NH_2_ 100 Å HILIC column (250×2.00 mm, 5 µ) (Phenomenex, Torrance, California) was used. The mobile phase flow rate was 150 µL/min, and a gradient of two solvents was used for elution. Solvent A was a mixture composed of 95% 20 mM ammonium acetate, 20 mM ammonium hydroxide in HPLC grade water buffered at pH = 9.4 with 5% HPLC grade acetonitrile. Solvent B was pure HPLC grade acetonitrile. These were used to construct the following 40 min gradient elution profile: *t*) 0 min, 15% solvent A, 85% solvent B; *t*) 15 min, 100% solvent A, 0% solvent B; *t*) 28 min, 100% solvent A, 0% solvent B; *t*) 30, 15% solvent A, 85% solvent B; *t*) 40, 15% solvent A, 85% solvent B. Separations were performed with the column temperature set at 10°C. For negative mode analyses, a Synergi 4 µ Hydro-RP 80 Å C18 column (150×2.00 mm, 4 µ) (Phenomenex, Torrance, California) was used. The mobile phase flow rate was 200 µL/min, and a gradient of two solvents was again used for elution. Solvent A consisted of a mixture containing 97% 11 mM tributylamine and 15 mM acetic acid in HPLC grade water and 3% HPLC grade methanol. Solvent B was pure HPLC grade methanol. These were used to construct the following 50 min gradient elution profile: *t*) 0 min, 100% solvent A, 0% solvent B; t) 5 min, 100% solvent A, 0% solvent B; *t*) 10 min, 80% solvent A, 20% solvent B; *t*) 15, 80% solvent A, 20% solvent B; *t*) 30, 35% solvent A, 65% solvent B; *t*) 33, 5% solvent A, 95% solvent B; *t* ) 37, 5% solvent A, 95% solvent B; *t*) 38, 100% solvent A, 0% solvent B; *t*) 50, 100% solvent A, 0% solvent B. Separations were performed with the column temperature maintained at 25°C. For both positive and negative mode analyses, the eluent was introduced directly into the MS for ion detection.

### Mass Spectrometric Detection Parameters

Samples were introduced into the electrospray ionization (ESI) chamber of a Finnigan TSQ Quantum Discovery Max triple quadrupole MS (Thermo Electron) through a 0.1 mm internal diameter fused silica capillary after delivery by HPLC as described above. The spray voltage for the ESI source was set to 4500 V, if detection occurred in positive ion mode If detection occurred in negative mode, the spray voltage for the ESI source was set to 3000 V. Nitrogen was used as the sheath gas (40 psi), and the inlet capillary temperature was 290°C. Argon was used as the collision gas at a pressure of 1.5 mTorr. Samples were analyzed using selected reaction monitoring (SRM) with the scan time for each SRM set to 0.05 s and a scan width of 1 *m/z*. Full selected reaction monitoring (SRM) detection parameters for most compounds have been previously reported by Rabinowitz and coworkers [33].

### Metabolomics Data Handling

Raw files from each sample were processed using the Xcalibur MS analysis software (Thermo Electron, Waltham, Massachusetts). The peak for each detected metabolite was manually integrated to determine the peak area using the Quan Browser function of Xcalibur. The integration values were then directly entered into a Microsoft Excel spreadsheet where they were normalized to the mass of tissue extracted. Since inter-day variability in the ion counts for metabolites was observed due to MS sampling variation, the treated samples were compared only to the control samples run on the same day. This comparison consisted of averaging the ion counts for the duplicate measurements of a metabolite from a treated sample on a given day and then dividing this number by the average of the duplicate measurements of the same metabolite from the control sample obtained on the same day to generate the fold change for the compound. The fold changes for the metabolite from each of the three days were then averaged and displayed in heat map format and clustered using the freely available Cluster 3.0 (www.falw.vu/~huik/cluster.htm) and Java Treeview [34] data analysis packages.

### Statistical Analyses

A one-way ANOVA (GraphPad Prism) was performed to detect statistical differences within all biochemical assays. Differences within the ANOVA were determined using a Tukey post hoc test. All data are reported as means ± SEM. For statistical analysis of data acquired using the aforementioned mass spectrometry techniques, the p value for each metabolite fold change was obtained by performing an F-test using the data analysis tool pack in Microsoft Excel. The variance in the fold changes for the metabolites in the treatment group was compared to a set of standardized fold changes for the metabolites from the control group that was generated by taking the intra-day ratio of the ion counts from the duplicate measurements of the control.

## Results

### INS-1E and 832/13 Rat Insulinoma Cells Display Similar Sensitivities to Pro-inflammatory Cytokines or Genuine Apoptotic Inducers

**Figure 1:**
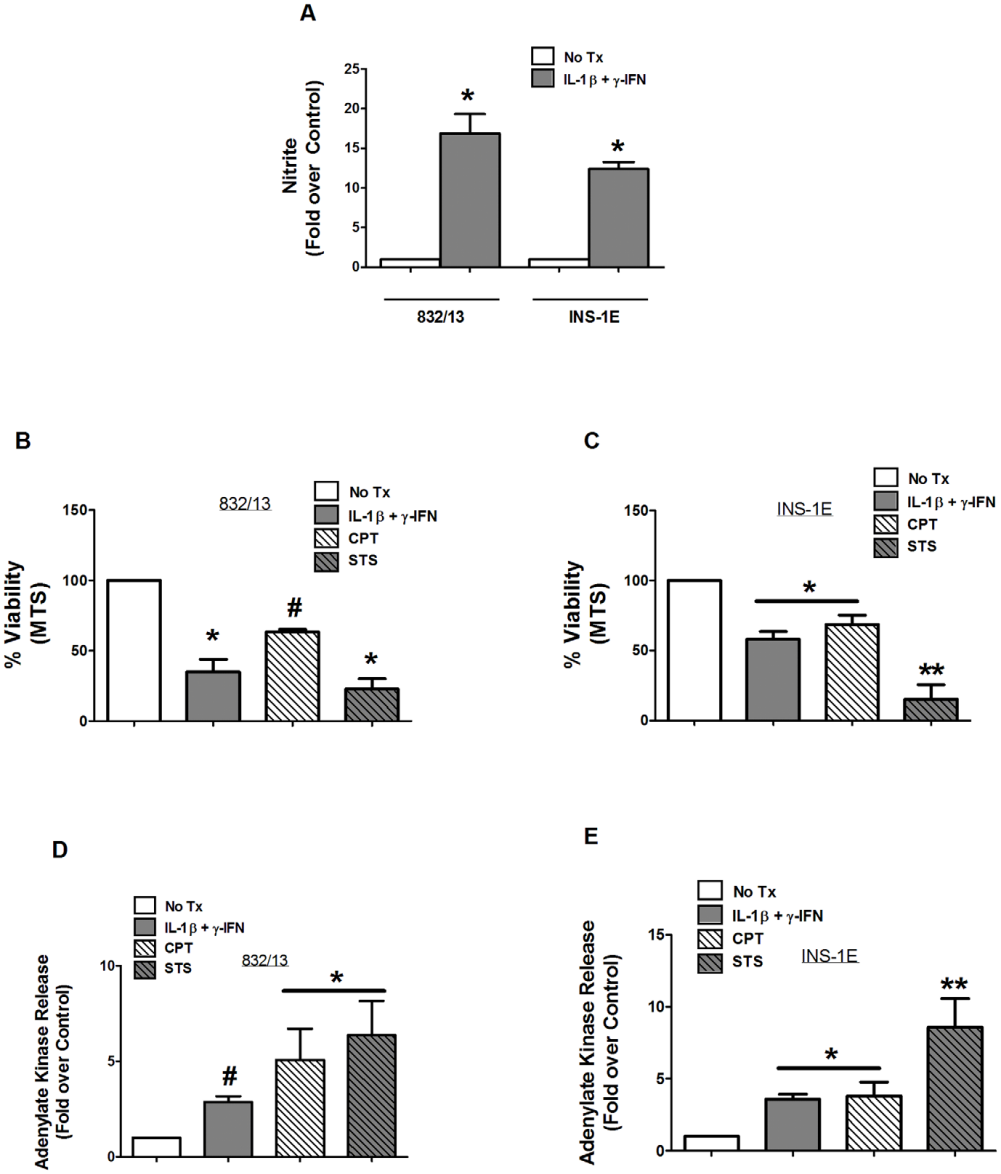
INS-1E and 832/13 rat insulinoma cells both display sensitivity to pro-inflammatory cytokines and genuine apoptotic inducers. 832/13 and INS-1E cells were exposed to either media alone (No Tx), 1 ng/mL IL-1β+100 U/mL γ-IFN for 18 h, 2 µM camptothecin for 6 h or 1 µM staurosporine for 6 h. *A*. Nitrite accumulation was measured by the Griess assay. Cell viability was measured by both MTS assay (*B* and *C*), and adenylate kinase assay (*D* and *E*). Data are means ± SEM for three individual experiments. #, p = 0.05 vs. control; * p<0.05 vs. control; **, p<0.01 vs. control.

**Figure 2 pone-0022485-g002:**
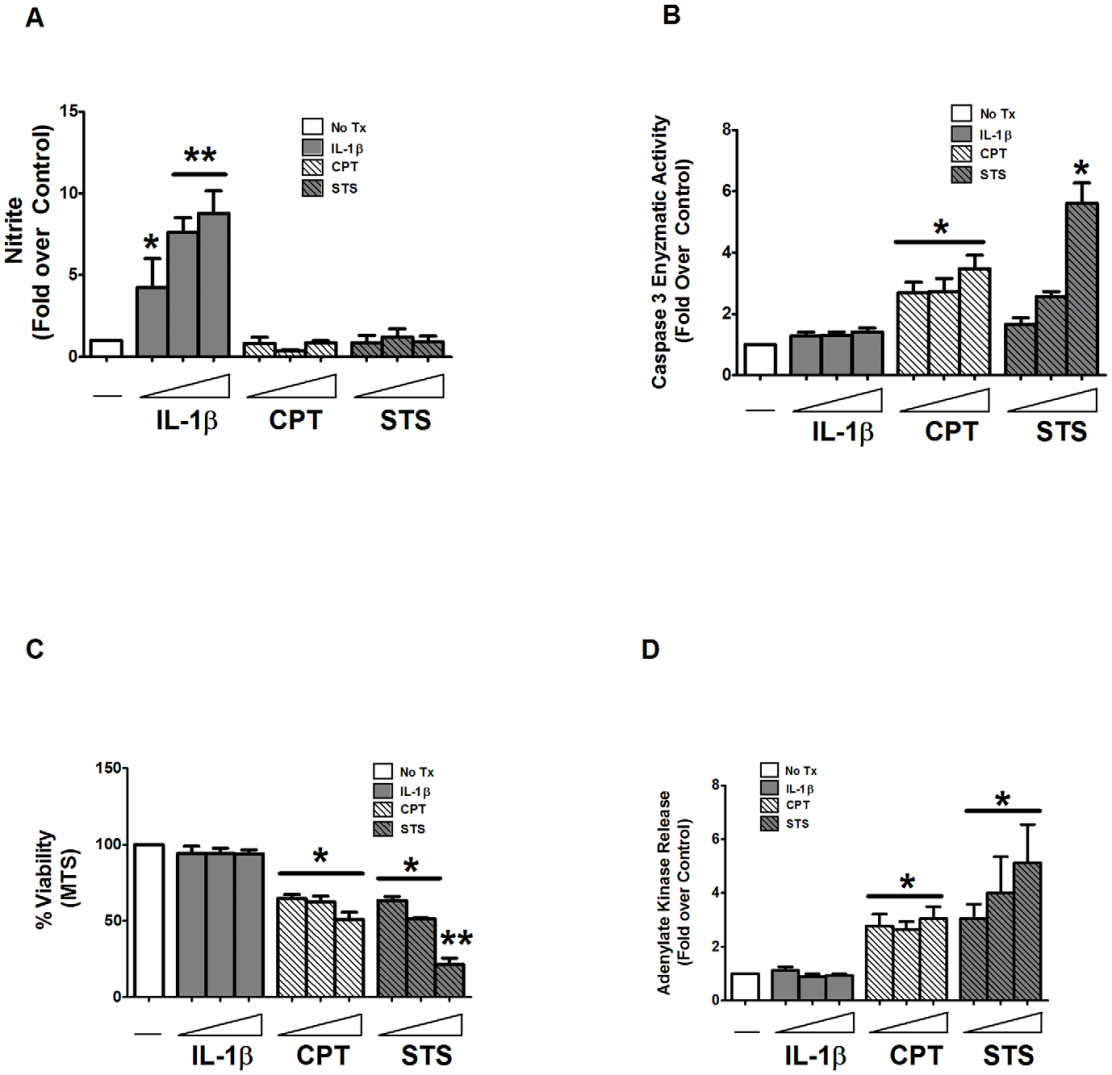
Pro-inflammatory cytokines have a dose-dependent effect on INS-1-derived cell lines. 832/13 cells were treated with standard culture media as a control (No Tx), increasing concentrations of IL-1β (0.01, 0.1, and 1 ng/mL) for 18 h, camptothecin (0.5, 1, and 2 µM) for 6 h or staurosporine (0.25, 0.5, 1 µM) for 6 h. Nitrite accumulation (*A*), caspase 3 enzyme activity (*B*), cell viability via MTS (*C*) or adenylate kinase release (*D*) were measured. Data represent means ± SEM for three individual experiments. *, p<0.05 vs. control; **, p<0.01 vs. control.

832/13 [26] and INS-1E [27] cells were derived from the original INS-1 population [35] by distinct selection protocols. It was hypothesized previously that G418 selection of 832/13 cells from the parental INS-1 population might impair the sensitivity of this cell line to the effects of IL-1β and γ-IFN [13]. To directly test this possibility, we investigated the sensitivity of the two aforementioned INS-1-derived cell lines to IL-1β and γ-IFN as well as to bona fide inducers of apoptosis. Exposure to 1 ng/mL IL-1β+100 U/mL γ-IFN (18 h) generated 16.8-fold (832/13) and 9.9-fold (INS-1E) nitrite accumulation in the media (Figure 1A), which correlated with 66.7% and 42% decreases in viability as measured by mitochondrial reduction of [3-(4,5-dimethylthiazol-2-yl)-5-(3-carboxymethoxyphenyl)-2-(4-sulfophenyl)-2H-tetrazolium (MTS)] (Figure 1B and C). In addition, release of adenylate kinase (ADK) into the media, a second independent measure of viability [11], confirmed the results seen with MTS assays (Figure 1D and E). From these results, it is clear that both of these INS-1-derived cell lines display robust sensitivity to IL-1β+γ-IFN, which has not been reported previously. Next, we selected two different inducers of apoptosis based on their known mechanisms of action: camptothecin (CPT), a topoisomerase I inhibitor, and staurosporine (STS), a general protein kinase inhibitor. Using 2 µM CPT and 1 µM STS, we observed similar losses in viability in both 832/13 and the INS-1E cell lines; however, exposure to these compounds did not lead to the production of nitrite (Figure 2A), demonstrating a key distinction in mechanism of killing between pro-inflammatory cytokines and apoptotic inducers, consistent with previous reports [9], [11]. Thus, 832/13 and INS-1E cells display comparative sensitivities to pro-inflammatory cytokines and apoptotic agents, despite distinct selection strategies used to obtain these clonal cell lines.

It has been postulated that the dose of cytokines might determine which cell death pathway is preferentially activated [13]. To test this hypothesis, we examined the effects of adding three different concentrations of IL-1β (0.01, 0.1, and 1 ng/mL) to both 832/13 and INS-1E cell lines to determine whether or not the concentration of pro-inflammatory cytokines selects for apoptosis or necrosis in β-cells. While there was a clear dose-dependent increase in nitrite production with IL-1β (Figure 2A), showing that the cells are capable of responding to different doses of cytokines, there was no significant caspase-3 enzymatic activity at any concentration tested (Figure 2B). By contrast, as concentrations of STS were increased, there was a corresponding dose-dependent enhancement in caspase-3 activity (Figure 2B); CPT also augmented caspase-3 activity similarly at all concentrations examined in this study (Figure 2B). We noted that pro-inflammatory cytokines and bona fide inducers of apoptosis all led to cellular demise as measured by MTS and ADK (Figure 2C and D). Our results are therefore consistent with a previous report in human islets where doses of IL-1β similar to those used here did not induce apoptosis [36] and with other existing evidence that nitric oxide accumulation prevents activation and activity of caspases [37], [38], [39].

**Figure 3:**
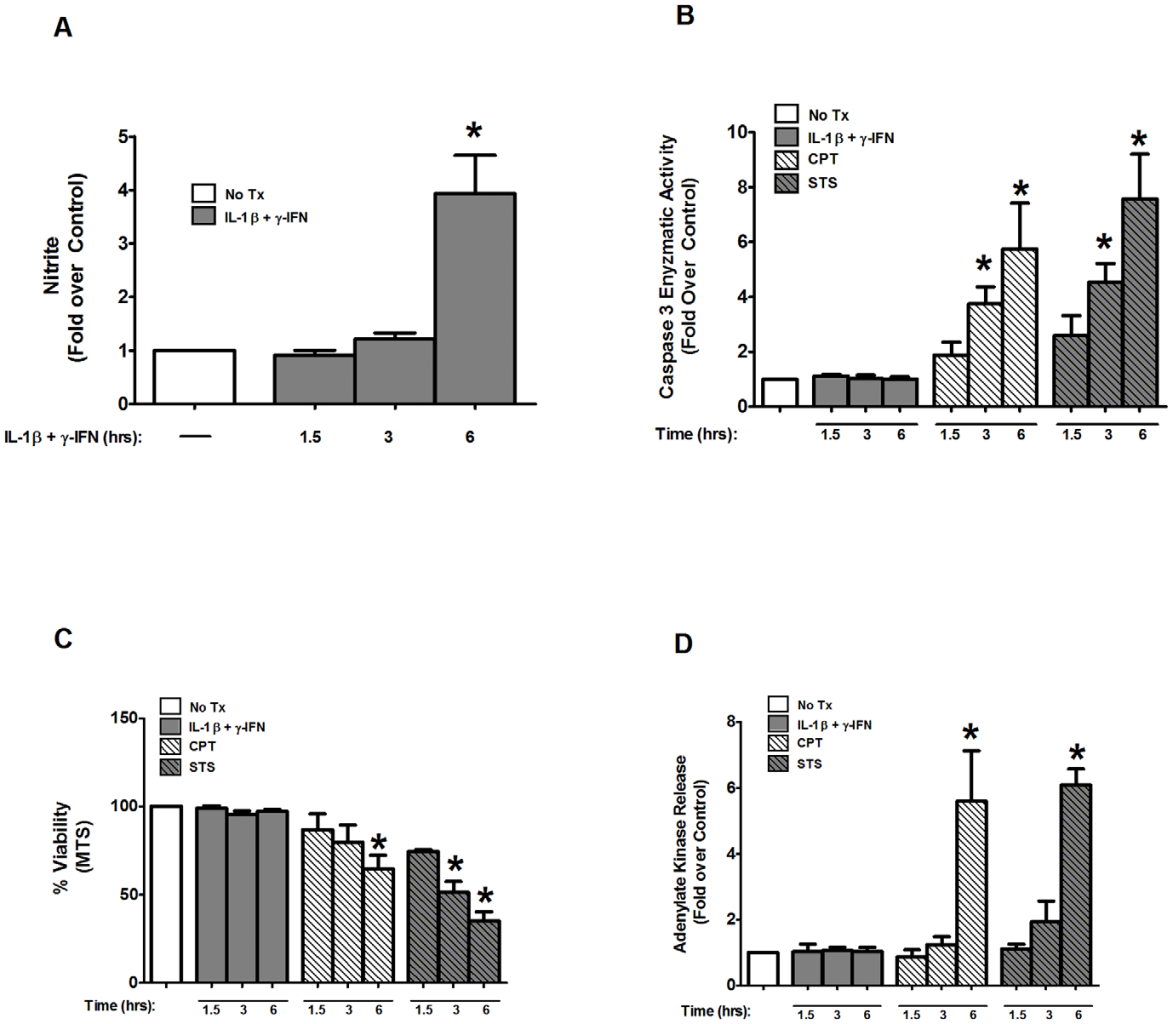
Time-dependent sensitivity of INS-1-derived cell lines in response to pro-inflammatory cytokines and bona fide apoptotic inducers. 832/13 cells were treated with media alone (No Tx), 1 ng/mL IL-1β plus 100 U/mL γ-IFN, 2 µM camptothecin or 1 µM staurosporine for 1.5, 3, and 6 h. Measurements of nitrite (*A*), caspase 3 enzyme activity (*B*), cellular viability via MTS (*C*) and adenylate kinase (*D*) were analyzed. Data are means ± SEM for three individual experiments. *, p<0.05 vs. control.

Because apoptosis occurs rapidly in response to a particular stimulus, we measured the temporal aspects of pro-inflammatory cytokines versus inducers of apoptosis in both 832/13 and INS-1E β-cell lines over 6 h. Note that while nitrite accumulates at 6 h in the presence of IL-1β+γ-IFN (Figure 3A), which demonstrates clear evidence of cytokine action, there is no attendant increase in caspase-3 enzyme activity (Figure 3B). Conversely, CPT and STS induced a time-dependent 5.7-fold and 7.6-fold increase in caspase-3 enzyme activity at 6 h (Figure 3B). In all cases of true apoptotic induction, augmentation of caspase-3 activity correlated with losses in viability, as measured by both the MTS and ADK assays (Figure 3C and D). We note that while a 6 h exposure to IL-1β was insufficient to impair viability (Figure 3C and D) despite increased nitrite production, this timing was adequate to reduce glucose-stimulated insulin secretion (JJC, unpublished observation). Our current observations are therefore consistent with studies in both rat and human islets [9], [11].

### Metabolic profiling by Tandem Mass Spectrometry Reveals Distinct Metabolite Responses of Cells Exposed to Pro-inflammatory Cytokines vs. the Apoptotic Inducer Camptothecin

**Figure 4:**
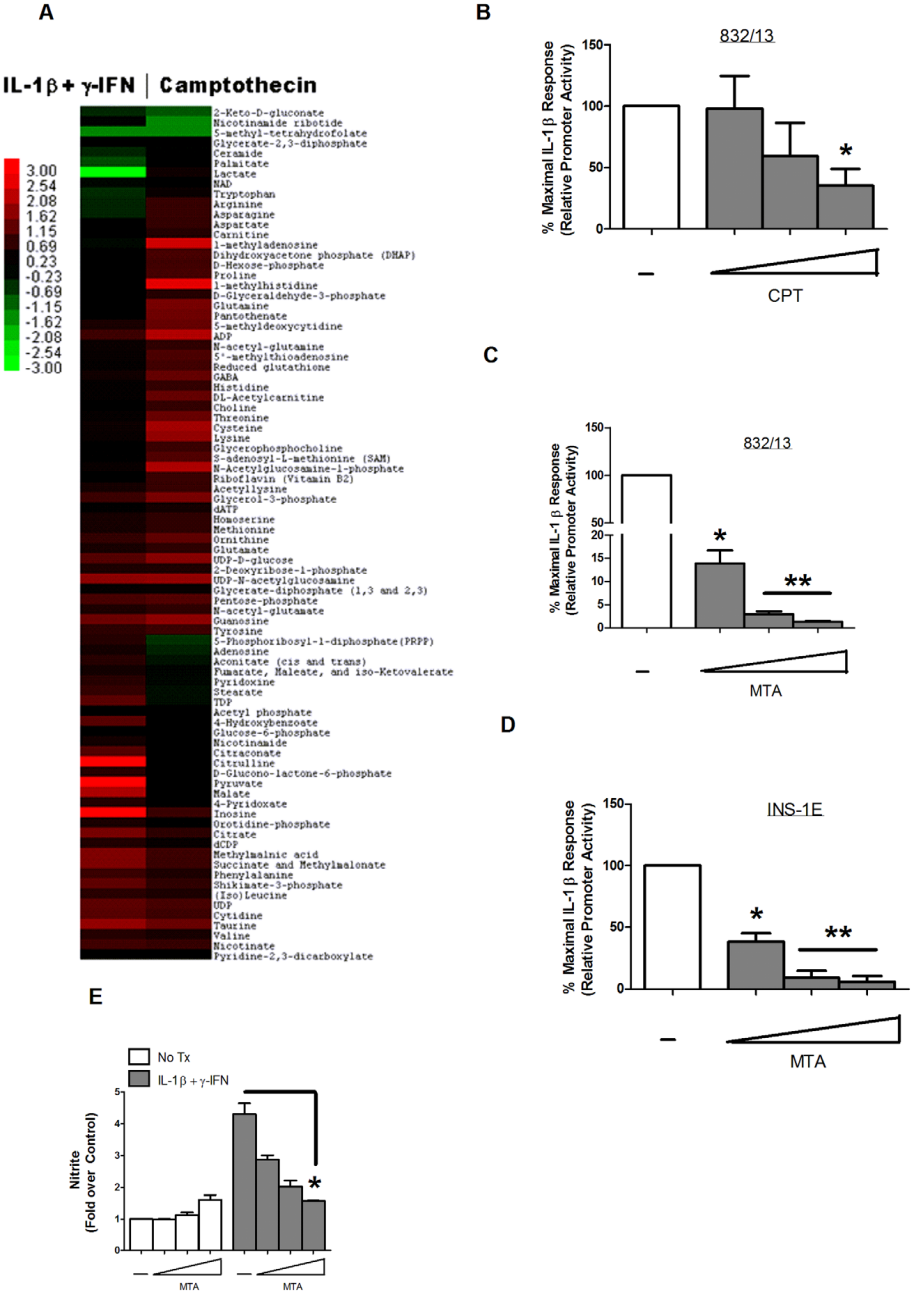
Metabolic profiling by tandem mass spectrometry reveals distinct metabolite signatures of cells exposed to pro-inflammatory cytokines vs. the apoptotic inducer camptothecin. *A*. 832/13 cells were exposed to 1 ng/mL IL-1β+100 U/mL γ-IFN or 2 µM camptothecin for 6 h. Metabolite concentration was measured by mass spectrometry with values displayed as fold change relative to control. The represent values given in log_2_, so that “3.00” is a minimum of an 8-fold increase, while “−3.00” is a minimum of an 8-fold decrease. The red color indicates a metabolite increase in the treatment condition relative to an untreated control, while the green color represents a decrease relative to control. The *p values* for all metabolites are given in Tables S1 and S2. *B*. 832/13 cells were transduced with recombinant adenovirus expressing a luciferase reporter gene driven by three NF-κB response elements (NF-κB-luc). After 24 h, cells were cultured in increasing concentrations of camptothecin (0.5, 1, 2 µM) for 1 h followed by a 4 h incubation with 1 ng/mL IL-1β. Luciferase activity was measured using cell lysates. *C–D*. 832/13 and INS-1E cells were transduced with the aforementioned NF-κB-luc virus. After 24 h, these cells were then exposed to increasing concentrations of MTA (1, 2, and 4 mM) for 1 h, followed by a 4 h exposure to 1 ng/mL IL-1β. After 4 h, luciferase activity was measured. *E*. 832/13 cells were cultured in the presence or absence of increasing concentrations of MTA (0.5, 1, and 2 mM) for 1 h, then switched to either media alone (No Tx) or 1 ng/mL IL-1β+100 U/mL γ-IFN for 6 h, followed by measurement of nitrite accumulation in the media. *, p<0.05; **, p<0.01.

While pro-inflammatory cytokines and genuine inducers of apoptosis are both capable of decreasing viability (See Figures 1–3 and [9], [11]), we hypothesized that they do so via different mechanisms. Thus, to further compare these cellular death effectors, we used a metabolomic tandem mass spectrometric approach to investigate metabolite changes that occur in response to either IL-1β+γ-IFN or CPT in 832/13 cells. The rationale for this comparison was that since nitric oxide accumulation produces DNA damage [40] via production of micromolar amounts of intracellular nitric oxide [4], [5], while CPT triggers apoptosis through DNA damage generated by topoisomerase inhibition [41], different metabolic pathways would be used depending on the mechanism of cell death. Concentration changes in ∼90 metabolites generated by these distinct stimuli measured relative to their respective control groups are displayed in heatmap form (Figure 4A), and these data reveal a discrete pattern of metabolites that change in a stimulus-specific pattern (e.g., IL-1β+γ-IFN vs. CPT).

For example citrulline, an end product of the inducible nitric oxide synthase reaction [42], accumulates approximately 400-fold (see Table S1); this is fitting with nitrite production observed upon cellular exposure to pro-inflammatory cytokines and thus serves as an internal control for cytokine efficacy (note that there is no increase in citrulline with CPT – see Table S2). Therefore, we believe that these data are sufficient to conclude that while both types of stimuli (i.e., cytokines and CPT) generate losses in cellular viability [see [9], [11] and Figures 1–3], the metabolic profile of each specific stimulus is visibly distinct.

Since CPT and IL-1β+γ-IFN produced discrete metabolic profiles (Figure 4A and Tables S1 and S2), we examined the possibility that metabolites altered during apoptosis were potentially interfering with the actions of pro-inflammatory cytokines. From the metabolomic data, it was noted that the 5′-deoxy-5′-methylthioadenosine (MTA) concentration was higher in cells treated with CPT than in those exposed to IL-1β+γ-IFN. MTA is involved in the biosynthesis of polyamines, a group of compounds that are associated with DNA synthesis and replication [43] and in controlling NF-κB activity through monomethylation of the p65 subunit [44]. To examine the effects of either CPT or MTA on NF-κB activity, we transduced 832/13 and INS-1E cells with a recombinant adenovirus that contains a luciferase reporter gene driven by multiple NF-κB elements (Figure 4B). IL-1β increased the activity of this reporter gene construct by 24-fold. CPT exposure, which promoted MTA accumulation (Figure 4A), decreased IL-1β-mediated induction of the NF-κB promoter activity by 39% (Figure 4B). To examine whether direct MTA addition has effects similar to that of CPT, we added exogenous MTA to both INS-1E and 832/13 cells and observed a dose-dependent decrease in the activity of the same NF-κB luciferase reporter construct in both cell lines (Figure 4C and D). In addition, direct MTA addition inhibited cytokine-mediated nitrite production (Figure 4E), which fits with its effects on NF-κB promoter activity. Thus, the current results are consistent with pro-inflammatory cytokines and apoptotic inducers activating distinct pathways in pancreatic β-cells and with MTA accumulation during apoptosis interfering with NF-κB activity.

### Molecular or Pharmacological Interference with NF-κB Activity Protects against Cytokine-Mediated Cell Death but not Apoptosis

**Figure 5:**
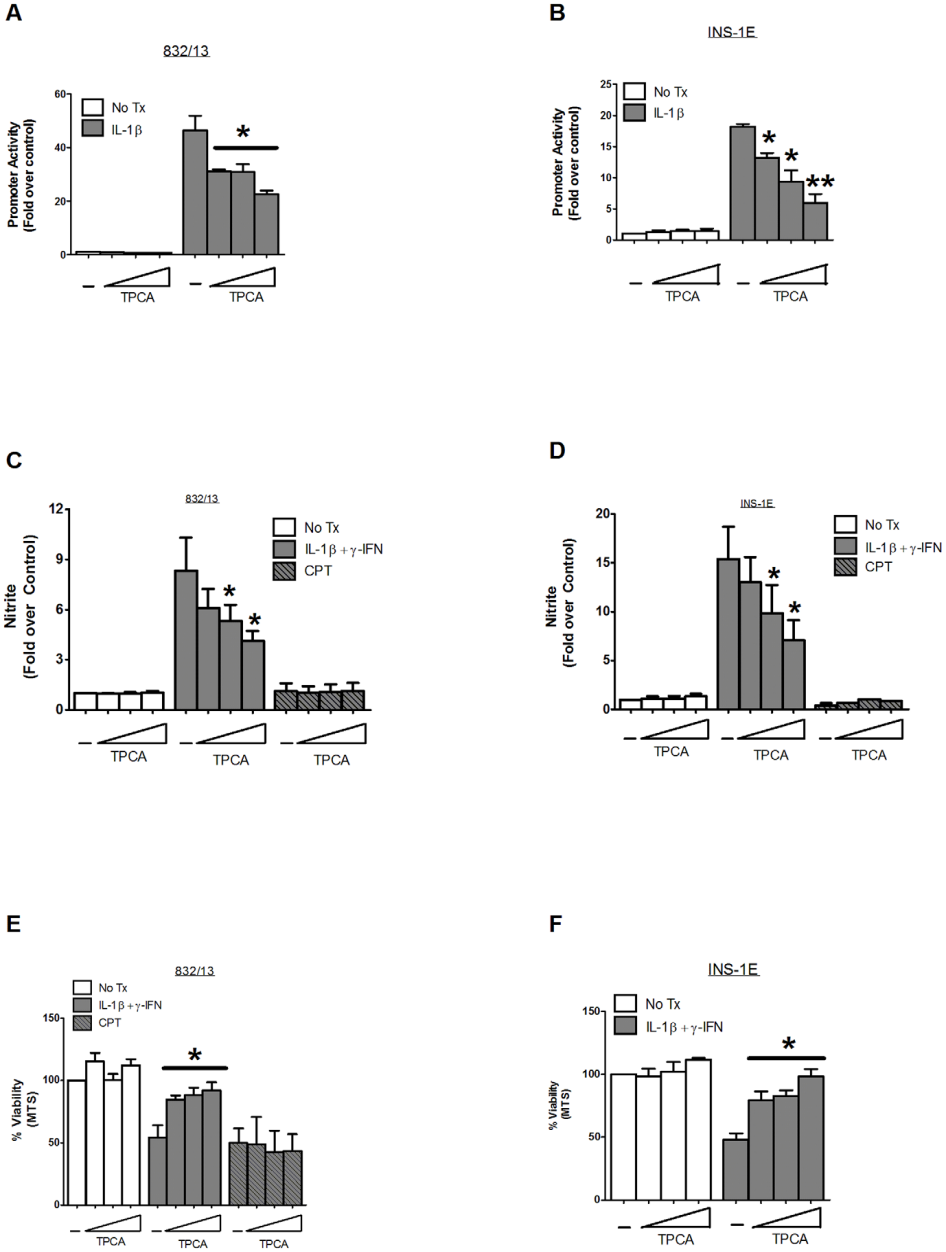
Pharmacological interference with NF-κB activity protects against cytokine-mediated cell death but not apoptosis. *A–B*. 832/13 and INS-1E cells were transduced with the NF-κB-luc virus for 24 h, followed by exposure to increasing concentrations of TPCA (0.5, 1, and 2 µM) for 1 h. Luciferase activity was measured following by a subsequent 4 h culture with 1 ng/mL IL-1β. Data are means ± SE for three independent experiments, performed in duplicate each time. *C–F*. 832/13 and INS-1E cells were pre-treated with increasing concentrations of TPCA (0.5, 1, and 2 µM) for 1 h, followed by a 4 h treatment with either 1 ng/mL IL-1β+100 U/mL γ-IFN or 2 µM camptothecin. Nitrite was measured using the Griess assay (*C* and *D*) and viability was measured by MTS assay (*E* and *F*). Data are representative of mean ± SEM for 3–4 individual experiments. *, p<0.05; **, p<0.01.

To further test the hypothesis that IL-1β+γ-IFN require discrete pathways to mediate their cytotoxic effects as compared to known inducers of apoptosis, we examined whether interference with the NF-κB signaling pathway impacted cellular responses to each of these stimuli. The IκB kinases (IKKs) control phosphorylation of both the inhibitory IκB proteins and the p65 subunit of NF-κB dimers and therefore serve as a convergence point for inflammatory stimuli, such as IL-1β that activate NF-κB [21]. Therefore, we used the pharmacological inhibitor of IKKβ, 2-[(Aminocarbonyl)amino]-5-(4-fluorophenyl)-3-thiophenecarboxamide (TPCA) to examine whether interference with a critical kinase in the NF-κB signaling pathway offered protection against pro-inflammatory cytokines or bona fide inducers of apoptosis. Using adenoviral delivery of an NF-κB driven luciferase reporter gene in 832/13 and INS-1E cells, we observed that TPCA inhibited cytokine-mediated induction of NF-κB activity (Figure 5A and B) and nitrite accumulation in a dose-dependent fashion (Figure 5C and D). Next, we measured the effects of TPCA treatment on viability in response to cellular exposure with either IL-1β+γ-IFN or CPT. In both 832/13 and INS-1E cells, TPCA provided significant protection as measured by the MTS assay (41 and 49% increase, respectively) against the pro-inflammatory cytokines but not to CPT (Figure 5E and 5F). Similar results were obtained with adenylate kinase release (data not shown). Our results are consistent with existing studies demonstrating anti-inflammatory effects for this compound in other systems [45] and reveal the involvement of β-cell IKKβin mediating the toxic effect of cytokines.

**Figure 6:**
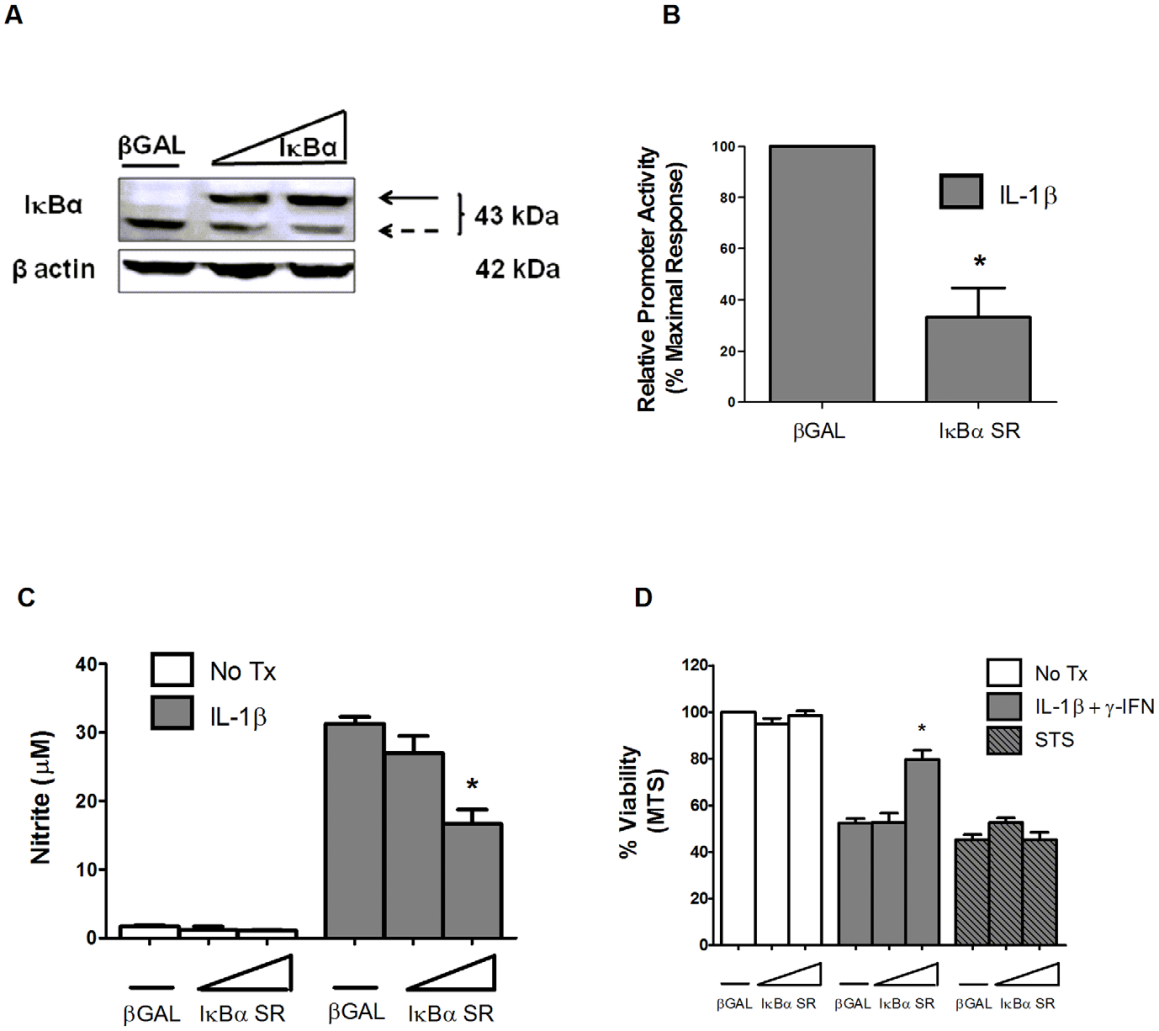
Overexpression of the IκBα super-repressor protects against pro-inflammatory cytokines but not genuine apoptotic induction. *A*. 832/13 cells were transduced with recombinant adenovirus expressing either β-galactosidase (βGal) or increasing concentrations of IκBα SR. *A*. Following 24 h incubation, whole cell lysates were immunoblotted for IκBα with β-actin as a loading control. Immunoblots represent 2 individual experiments. Note that HA-tagging of the IκBα SR causes it to migrate slower (top arrow) relative to the endogenous protein (bottom arrow). *B*. 832/13 cells were transduced with NF-κB-luc and either βGAL or the highest dose of the IκBα SR (shown in panel A) for 24 h. Cells were then treated with 1 ng/mL IL-1β for 4 h and luciferase activity was measured. Data are means ± SEM for 3 individual experiments. *C–D*. 832/13 cells were transduced with adenoviruses expressing either βGAL or increasing concentrations of IκBα. After 24 h, the cells were stimulated with either 1 ng/mL IL-1β or 1 µM STS for 6 h. Nitrite was measured via the Griess assay (C), and viability by MTS assay (D). Values represent means ± SEM from 4 individual experiments. *, p<0.05.

Next, we took a different approach to interfering with NF-κB activity by examining how a mutant version of IκBα, termed the IκBα super-repressor (IκBα SR), would impact cellular survival in response to pro-inflammatory cytokines and apoptotic inducers. The IκBα SR contains amino acid substitutions (S32A/S36A) that prevent degradation by the usual inflammatory signals that activate NF-κB [29]. Expressing this mutant IκBα protein using recombinant adenovirus in 832/13 cells increased total IκBα abundance (Figure 6A), which yielded a 69% decrease in IL-1β-mediated NF-κB promoter activity (Figure 6B) and a corresponding 52% decrease in nitrite production (Figure 6C); these observations correlate with a 34.3% increase in viability in 832/13 cells expressing the IκBα SR when compared to cells receiving β-galactosidase, a control for adenoviral transduction (Figure 6D). However, this maneuver was unable to prevent β-cell death in response to STS (Figure 6D). Taken together, these results are consistent with the requirement for NF-κB during cytokine-mediated β-cell death, but not to cellular demise in response to bona fide apoptotic induction.

### Manipulation of the Apoptosome Protects against Apoptosis but does not Impact Pro-inflammatory Cytokine-mediated Cell Death

**Figure 7:**
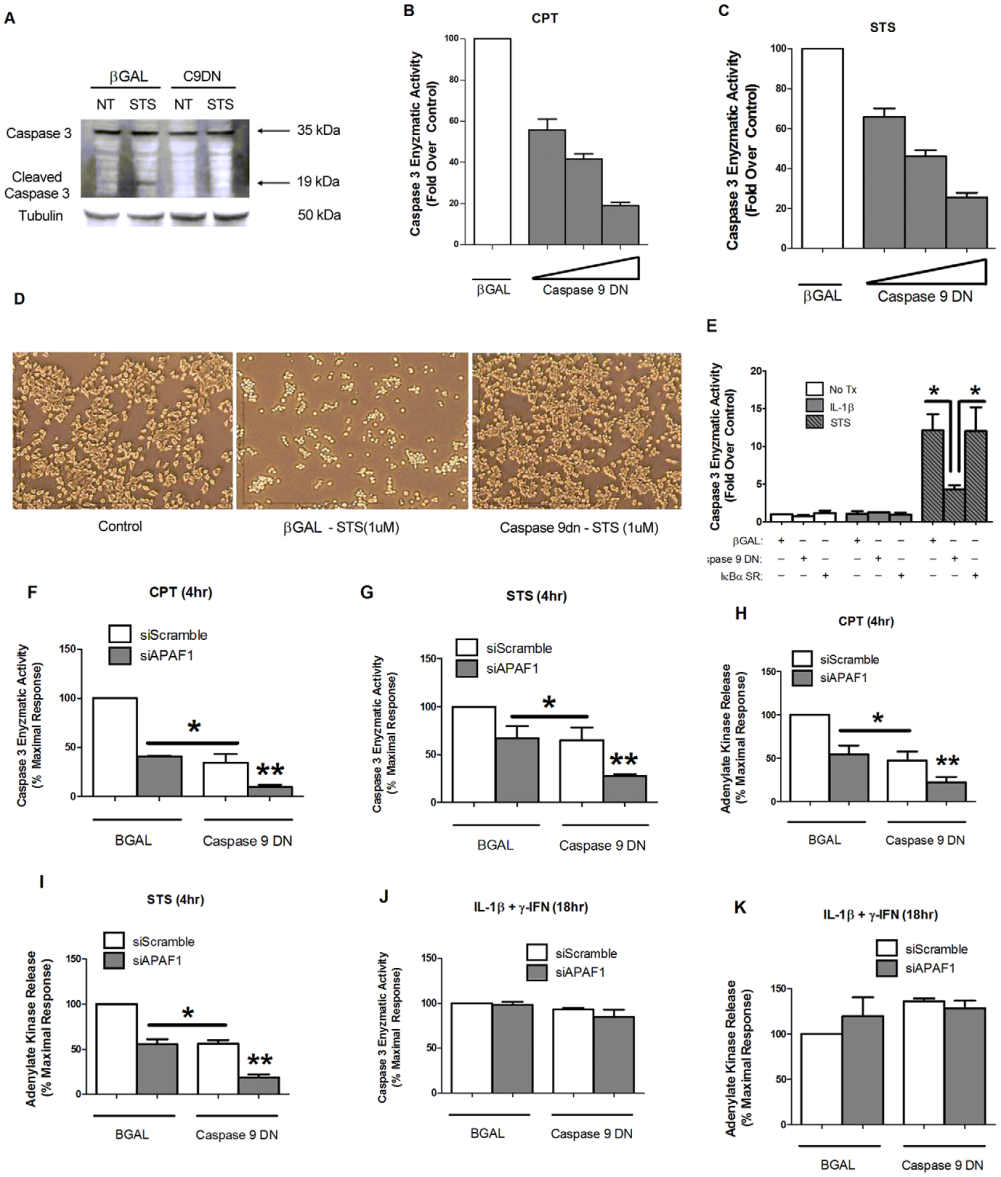
Manipulation of the apoptosome protects against apoptosis but does not impact pro-inflammatory of cytokine-mediated cell death. *A*. 832/13 cells were transduced either with a recombinant adenovirus expressing βGal or dominant-negative caspase 9 for 24 h followed by a change to media alone (control) or 1 µM staurosporine for 4 h. Whole-cell lysates were immunoblotted for caspase-3 with β-actin as a loading control. Top and bottom arrows indicate total and cleaved caspase-3, respectively. Immunoblots are representative of two independent experiments. *B* and *C*. 832/13 cells were transduced with either β-Galactosidase or increasing doses of the dominant-negative caspase-9 for 24 h, followed by 4 h stimulation with either 2 µM camptothecin (*B*) or 1 µM staurosporine (*C*). Cell lysates were analyzed for caspase-3 enzyme activity and expressed as percent of the maximal response. Data are presented as mean ± SEM for three separate experiments. *D*. The highest dose of Caspase-9 DN from (*B* and *C*) was then used to transduced 832/13 cells. 24 h post-transduction, cells were exposed to 1 µM staurosporine for 4 h, and then phase contrast images were acquired. *E*. Rat islets were transduced with the indicated adenoviruses; 72 h after adenoviral transduction, the islets were exposed to 10 ng/mL IL-1β or 1 µM staurosporine for 6 h and harvested for caspase-3 enzyme activity. *F-K*. 832/13 cells were transduced with recombinant adenoviruses expressing either βGAL or Caspase-9 DN. After 8 h of incubation with the indicated adenoviruses, cells were then transfected with siRNA duplexes representing either a scrambled control (siScramble) or specifically targeting the APAF-1 mRNA. 24 h after siRNA transfection, cells were treated with either 2 µM camptothecin (*F* and *H*) or 1 µM staurosporine (*G* and *I*) for 4 h or 1 ng/mL IL-1β+100 U/mL γ-IFN for 18 h (*J* and *K*). *, p<0.05; **, p<0.01.

Processing of caspase-9 from an inactive form to an active form occurs via interaction with a large protein complex called the apoptosome, which also consists of cytochrome c, dATP, and APAF-1 [46]. To address whether interfering with the activity of this complex protects against cytokine-mediated cell death or apoptosis or both, we used siRNA-directed suppression of APAF-1, expression of a dominant-negative caspase-9, or a combination of these approaches. The dominant-negative caspase-9 (C9DN) contains the caspase activation recruitment domain, but no catalytic domain, and thus is incorporated into the apoptosome but does not possess enzymatic activity and therefore does not process downstream pro-caspases [30]. Expression of the C9DN construct in 832/13 cells suppressed STS-induced cleavage of caspase-3 (Figure 7A) and also blunted the CPT- and STS-mediated increases in caspase-3 enzyme activity in a dose-dependent manner (81 and 74.4% decrease at the highest dose for CPT and STS, respectively– see Figure 7B and C); this maneuver has a pronounced impact on cellular structure as viewed by morphological analysis (Figure 7D). Moreover, the 12.1-fold increase in caspase-3 enzyme activity by STS in isolated rat islets was blocked by 64.5% in the presence of C9DN (Figure 7E); however, the IκBα super-repressor, despite having a strong repressive effect on cytokine-mediated NF-κB activation (Figure 6B), did not prevent STS-mediated increases in caspase-3 activity.

In a complementary approach, siRNA-mediated suppression of APAF-1 (89%) protected against CPT- and STS-mediated losses in viability (Figure 7F–I), but had no effect on cellular survival upon exposure to pro-inflammatory cytokines (Figure 7J and K). Additionally, expression of C9DN on the background of APAF-1 depletion was additive in protecting against bona fide inducers of apoptosis (Figure 7H and I). Despite being highly effective against killing by genuine inducers of apoptosis, extensive interference with apoptosome activity, via C9DN and siAPAF-1, afforded no protection against cytokine-mediated cell death (Figure 7J and K). We interpret these results to indicate that the pro-inflammatory combination of IL-1β+γ-IFN does not impair viability via an apoptosome requiring mechanism.

## Discussion

The recruitment of immune cells to the islets generates insulitis [47], an inflammatory response with an unknown origin that eventually produces losses in functional β-cell mass [48], [49]. It has been suggested that during this process, the pro-inflammatory cytokines IL-1β and γ-IFN induce apoptosis in the pancreatic β-cell [13], [50], [51]. However, several lines of evidence are not consistent with apoptosis as the major mechanism of β-cell death under these conditions. For example, pro-inflammatory cytokines increase the abundance of iNOS protein, leading to micromolar amounts of nitric oxide amassing within the β-cell [5], [52]; nitric oxide inhibits formation of the apoptosome complex required for activation of caspases [37], [39], [53]. In addition, biochemical and morphological evidence from direct comparisons of pro-inflammatory cytokines and bona fide inducers of apoptosis reveal differences in cellular death mechanisms activated by these stimuli [9], [11]. Furthermore, overexpression of the anti-apoptotic kinase Akt or depletion of the pro-apoptotic protein Bax provides protection against true apoptosis, but not against pro-inflammatory cytokines [11]. Finally, HMGB1, an immunological activating protein [23], is released in β-cells exposed to IL-1β, but not to the topoisomerase inhibitor CPT or the general protein kinase inhibitor STS (both genuine inducers of apoptosis) [9]; activation of the immune system by necrotic release of immuno-stimulatory molecules is also consistent with the insulitis and immune cell recruitment to pancreatic islets that leads to T1DM.

In the present study, we tested the hypothesis that true apoptosis is distinguishable from cellular death of pancreatic β-cells in response to pro-inflammatory cytokines. Several novel observations emerged from the current endeavor: 1) Despite different selection strategies from the parental INS-1 population, the INS-1-derived cell lines 832/13 and INS-1E were shown to be equally sensitive to killing by IL-1β+γ-IFN and genuine apoptotic stimuli, making them excellent models in which to precisely delineate β-cell death pathways. 2) Discrete metabolic signatures are produced by pro-inflammatory cytokines as compared to CPT, a bona fide apoptotic inducer, as measured by tandem mass spectrometry based metabolomic approach. 3) The metabolite 5′-methylthioadenosine (MTA) is increased when cells are exposed to CPT but not IL-1β+γ-IFN; direct addition of MTA to β-cell lines inhibits cytokine-mediated NF-κB activity and nitrite production. 4) Inhibition of IKKβ or overexpression of an IκBα super-repressor protein blunted cytokine-induced nitric oxide production and provided protection against losses in β-cell viability; no protection against CPT-mediated apoptosis was observed using either of these maneuvers. 5) Manipulation of the apoptosome, via siRNA-mediated suppression of APAF-1 or a dominant-negative form of caspase-9, protected against cell death induced by CPT or STS, but not IL-1β+γ-IFN.

We note that the concentrations of the majority of metabolites measured via mass spectrometry increased in both treatment conditions. However, this should not necessarily be taken as an indication that the dying cells are more metabolically active than the corresponding control since concentration measurements alone are not sufficient to determine whether a build-up of a particular metabolite is due to an increase in biosynthesis *or* to a decrease in usage of that compound. Nonetheless, the differences in metabolite signature (Figure 4) between pro-inflammatory cytokines and bona fide inducers of apoptosis fit with the distinction in biochemical measurements made in this study and in previous studies [9], [11].

An earlier report proposed a mitochondrial or intrinsic activation of apoptosis in pancreatic β-cells in response to pro-inflammatory cytokines [13]. However, no control for genuine apoptosis was used in those or other recent studies [12], [13]. In addition, we note here that compromised mitochondrial function and release of cytochrome c may not be sufficient for or indicative of apoptosis [54]. For example, in photodynamic treatment of cancer cells, reactive oxygen species (ROS) accumulate, mitochondrial function is compromised, cytochrome c is released, yet necrosis occurs [55], [56]. In addition, eliminating the ROS using antioxidants is protective, while inhibiting ROS scavenging enzymes, such as superoxide dismutase, enhances cell death [57]. This mechanism is reminiscent of β-cell death in response to cytokines: ROS levels accumulate [58], [59], mitochondrial function is impaired [60], ATP levels decrease [11], [61], and no detectable markers of apoptosis are present despite large increases in cell death [9], [11]; ROS destroying enzymes also provide protection against pro-inflammatory cytokines in pancreatic β-cells [62]. Thus, the data do not seem to support apoptosis as the predominant form of β-cell death in response to pro-inflammatory cytokines.

Additionally, prior studies using *in vivo* models of autoimmune-mediated β-cell death (e.g., NOD mice with cyclophosphamide accelerated diabetes) found lack of caspase-3 activation in β-cells, despite the presence of IL-1β [63]. Moreover, transfusion of β-cells made apoptotic *ex vivo* prevents Type 1 diabetes in NOD mice [64]. Also, despite DNA strand breaks (which are not by themselves markers of apoptosis [18]) in the islets of BB rats, there is no evidence of apoptosis leading to β-cell destruction [10]. Furthermore, transgenic expression of the anti-apoptotic protein Bcl-2 does not prevent losses in pancreatic β-cell mass in animals with autoimmune forms of diabetes [65]. Collectively, the aforementioned *in vitro* and *in vivo* data, as well as the results in this report, are all consistent with inflammation leading to necrotic cell death; this fate of pancreatic β-cells would likely generate a strong immune response targeted to the insulin-producing cells, which does occur in individuals with autoimmune forms of diabetes, while the available evidence is in favor of apoptosis being more likely to have the opposite effect [18], [19], [23], [66].

**Figure 8:**
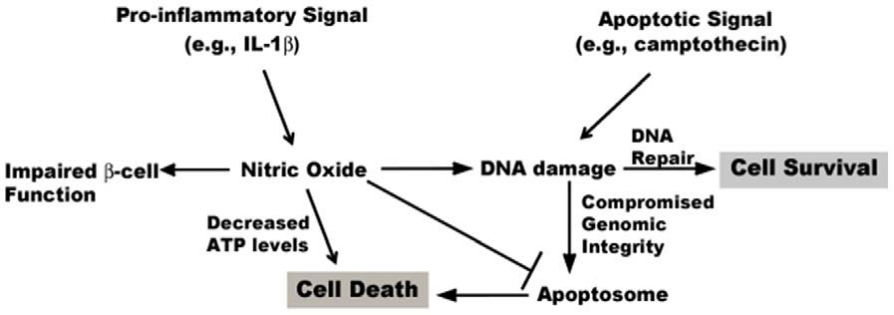
Cell death pathways activated in pancreatic β-cells in response to pro-inflammatory or apoptotic stimuli. The combination of IL-1β+γ-IFN generates massive quantities of nitric oxide within the pancreatic β-cell which impairs β-cell function, decreases intracellular ATP levels, and inhibits apoptosome formation and/or activity, leading to cellular death by necrosis. Apoptotic signals, such as camptothecin, lead to DNA damage without nitric oxide accumulation, which signals apoptosome formation. Nitric oxide inhibits apoptosome activity but also damages DNA, which the cell attempts to repair. If the damaged DNA cannot be repaired *and* nitric oxide levels decrease sufficiently to allow apoptosome formation, a subset of β-cells may undergo apoptosis provided adequate ATP levels are present.

Does this mean that apoptosis is absolutely not represented during pro-inflammatory cytokine-mediated β-cell death? We suspect that there might be specific, albeit limited, circumstances in which a subset of pancreatic β-cells may be able to undergo apoptosis in response to cytokine-mediated damage. For instance, during the acute (2–24 h) period of cytokine exposure, gene expression changes are occurring rapidly that lead to the buildup of inflammatory mediators, such as nitric oxide and prostaglandins, that are eventually toxic to pancreatic β-cells [67]. During this period, no detectable markers of apoptosis are apparent (current study and see [9], [11], [68]). However, if nitric oxide levels decrease, presumably to amounts no longer able to inhibit the apoptosome [37], [39], apoptosis may proceed if cellular DNA is unable to be repaired [68]. Importantly though, sufficient ATP levels must be present for caspase-mediated cell death, otherwise necrosis will prevail [11], [69]. Therefore, it is quite possible that pro-inflammatory cytokines preferentially induce a programmed necrotic cell death (the concept of which has been reviewed recently [70]) with the possibility for a secondary apoptotic event only under specific conditions (Figure 8). Indeed, the necrotic program initiated by pro-inflammatory cytokines may actually actively suppress apoptosis via the mechanisms described above, much like apoptotic signaling proteins can prevent necrosis [71], [72], [73]. We also note that the role of autophagy, another regulated form of cellular demise that can be linked with inflammation in response to γ-IFN [74], is largely unexplored in the pancreatic β-cell. However, since autophagy may play a role in maintenance of islet function during insulin resistance [75] as well as islet graft malfunction [76], its role in both major forms of diabetes should be considered alongside programmed cellular necrosis.

In summary, we demonstrated here that induction of apoptosis by CPT and STS is rapid (Figures 1–3), consistent with general descriptions of this cellular death mechanism (for reviews, see [15], [77]). Furthermore, interference with the apoptosome by siAPAF-1 and caspase-9 dominant-negative approaches protected against known apoptotic agents, but not pro-inflammatory cytokines. On the other hand, established strategies for blocking NF-κB activity were highly effective against pro-inflammatory cytokines but not apoptotic induction (Figures 5 and 6). Taken together, these observations fit with the distinct metabolic profiles generated by these two different β-cell death-inducing stimuli (See heatmap – Figure 4A and Tables S1 and S2). Thus, interfering with signaling to NF-κB through IKKβ or the IκBα super-repressor (see Figures 5 and 6 and [78]) offered robust protection against cytokine-mediated β-cell death, while maneuvers that impact apoptosis, such as constitutively-active Akt or siRNA-mediated suppression of Bax [11], or manipulation of the apoptosome (Figure 7) provide protection against authentic apoptotic signals. In addition, it is likely that some of the inflammatory actions of IL-1β are mediated through the IKKs [79]. Therefore, inhibition of the IKKs, such as with TPCA (Figure 5) or by peptide inhibitors [80] is a reasonable strategy to offset death and dysfunction mediated by pro-inflammatory cytokines. Based on results presented herein and those of other extant studies, we conclude that β-cell fate in response to pro-inflammatory is unlikely to proceed through an apoptotic pathway.

## Supporting Information

Table S1 (DOCX)

Table S2 (DOCX)
