# Supplementary material for: Pancreatic β-Cell Death in Response to Pro-Inflammatory Cytokines Is Distinct from Genuine Apoptosis
Source: PLoS One. 2011 Jul 29;6(7):e22485. doi: 10.1371/journal.pone.0022485 (PMC3146470; doi:10.1371/journal.pone.0022485)
Supplement: Table S1 — (DOCX) [file pone.0022485.s001.docx]

| **Table S1: Metabolites Measured by MS** | | |
| --- | --- | --- |
| **Metabolite** | **Fold change in cells treated with IL-1β+γ-IFN Relative to NT** | **p value** |
| Citrulline | 431.17 | 2.65 x 10^-25^ |
| Inosine | 11.70 | 3.60 x 10^-7^ |
| Pyruvate | 7.58 | 2.03 x 10^-6^ |
| TDP | 2.14 | 8.55 x 10^-6^ |
| Lactate | -8.15 | 4.42 x 10^-5^ |
| Shikimate-3-phosphate | 2.20 | 7.20 x 10^-5^ |
| Malate | 3.78 | 1.38 x 10^-5^ |
| Taurine | 3.18 | 1.75 x 10^-4^ |
| UDP-N-acetylglucosamine | 2.98 | 2.12 x 10^-4^ |
| Methylmalnic acid | 2.78 | 3.95 x 10^-4^ |
| 4-Hydroxybenzoate | 2.10 | 4.16 x 10^-4^ |
| Succinate and Methylmalonate | 2.75 | 4.48 x 10^-4^ |
| Citrate | 2.65 | 5.49 x 10^-4^ |
| Citraconate | 2.06 | 6.52 x 10^-4^ |
| N-Acetylglucosamine-1-phosphate | 1.21 | 1.28 x 10^-3^ |
| D-Glyceraldehyde-3-phosphate | 1.00 | 1.65 x 10^-3^ |
| UDP-D-glucose | 2.05 | 2.25 x 10^-3^ |
| UDP | 2.18 | 2.68 x 10^-3^ |
| Nicotinamide ribotide | -1.16 | 3.75 x 10^-3^ |
| Pantothenate | -1.04 | 4.06 x 10^-3^ |
| dCDP | 1.44 | 5.13 x 10^-3^ |
| Glucose-6-phosphate | 1.09 | 6.46 x 10^-3^ |
| Glycerate-2,3-diphosphate | -1.09 | 8.09 x 10^-3^ |
| Nicotinate | 1.78 | 8.36 x 10^-3^ |
| Riboflavin (Vitamin B2) | 1.08 | 0.011 |
| Orotidine-phosphate | 1.38 | 0.011 |
| 5-methyl-tetrahydrofolate | -2.88 | 0.013 |
| 2-Deoxyribose-1-phosphate | 1.40 | 0.014 |
| 1-methyladenosine | -1.22 | 0.015 |
| Ceramide | -1.48 | 0.016 |
| Guanosine | 2.41 | 0.017 |
| Pyridine-2,3-dicarboxylate | 1.00 | 0.027 |
| Stearate | 1.64 | 0.033 |
| D-Glucono-lactone-6-phosphate | 1.52 | 0.036 |
| Dihydroxyacetone phosphate (DHAP) | -1.09 | 0.038 |
| Pyridoxine | 1.51 | 0.040 |
| Pentose-phosphate | 1.85 | 0.044 |
| dATP | 1.17 | 0.054 |
| Tyrosine | 1.57 | 0.058 |
| ADP | 1.74 | 0.063 |
| D-Hexose-phosphate | -1.05 | 0.066 |
| Asparagine | -1.47 | 0.093 |
| NAD | -1.19 | 0.106 |
| N-acetyl-glutamine | 1.23 | 0.120 |
| 1-methylhistidine | 1.00 | 0.134 |
| 5'-methylthioadenosine | 1.21 | 0.136 |
| 2-Keto-D-gluconate | -1.50 | 0.139 |
| Glycerate-diphosphate (1,3 and 2,3) | 1.19 | 0.140 |
| Aconitate (*cis* and *trans*) | 1.51 | 0.144 |
| Glycerol-3-phosphate | 1.70 | 0.148 |
| Methionine | 1.34 | 0.153 |
| Homoserine | 1.34 | 0.158 |
| Glutamate | 1.35 | 0.162 |
| Carnitine | -1.13 | 0.168 |
| Cytidine | 2.12 | 0.175 |
| Ornithine | 1.62 | 0.180 |
| Arginine | -1.47 | 0.204 |
| GABA | 1.29 | 0.228 |
| 4-Pyridoxate | 1.46 | 0.229 |
| Choline | 1.10 | 0.238 |
| Acetyllysine | 1.32 | 0.240 |
| 5-methyldeoxycytidine | 1.37 | 0.251 |
| Histidine | 1.17 | 0.259 |
| Cysteine | 1.25 | 0.264 |
| Proline | -1.06 | 0.270 |
| 5-Phosphoribosyl-1-diphosphate (PRPP) | 1.49 | 0.276 |
| N-acetyl-glutamate | 1.40 | 0.286 |
| Valine | 1.48 | 0.295 |
| Acetyl phosphate | 1.00 | 0.299 |
| DL-Acetylcarnitine | 1.17 | 0.316 |
| Reduced glutathione | 1.17 | 0.319 |
| Nicotinamide | 1.31 | 0.352 |
| Lysine | 1.21 | 0.354 |
| Glutamine | 1.03 | 0.361 |
| Tryptophan | -1.53 | 0.365 |
| Fumarate, Maleate, and iso-Ketovalerate | 1.31 | 0.369 |
| Phenylalanine | 1.72 | 0.417 |
| S-adenosyl-L-methionine | 1.13 | 0.436 |
| Threonine | 1.18 | 0.452 |
| (Iso)Leucine | 1.53 | 0.454 |
| Adenosine | 1.32 | 0.460 |
| Palmitate | -1.92 | 0.477 |
| Glycerophosphocholine | 1.10 | 0.478 |
| Aspartate | -1.15 | 0.484 |
|  | | |
