# Supplementary material for: Pancreatic β-Cell Death in Response to Pro-Inflammatory Cytokines Is Distinct from Genuine Apoptosis
Source: PLoS One. 2011 Jul 29;6(7):e22485. doi: 10.1371/journal.pone.0022485 (PMC3146470; doi:10.1371/journal.pone.0022485)
Supplement: Table S2 — (DOCX) [file pone.0022485.s002.docx]

| **Table S2: Metabolites Measured by MS** | | |
| --- | --- | --- |
| **Metabolite** | **Fold change in cells treated with CAMPTOTHECIN**  **Relative to NT** | **p value** |
| N-Acetylglucosamine-1-phosphate | 3.79 | 1.79 x 10^-5^ |
| Cysteine | 3.59 | 1.83 x 10^-5^ |
| 1-methylhistidine | 5.66 | 4.83 x 10^-5^ |
| D-Glyceraldehyde-3-phosphate | 1.50 | 5.23 x 10^-5^ |
| Pantothenate | 2.47 | 8.07 x 10^-5^ |
| 1-methyladenosine | 4.83 | 8.41 x 10^-5^ |
| Tryptophan | 1.23 | 3.52 x 10^-4^ |
| UDP-D-glucose | 2.90 | 4.35 x 10^-4^ |
| UDP-N-acetylglucosamine | 3.10 | 4.99 x 10^-4^ |
| Lactate | 1.26 | 6.76 x 10^-4^ |
| Methylmalnic acid | 1.73 | 9.15 x 10^-4^ |
| Succinate and Methylmalonate | 1.72 | 1.09 x 10^-3^ |
| 5-methyldeoxycytidine | 2.33 | 3.55 x 10^-3^ |
| 5-Phosphoribosyl-1-diphosphate (PRPP) | -1.61 | 4.71 x 10^-3^ |
| Nicotinamide ribotide | -2.92 | 5.70 x 10^-3^ |
| Shikimate-3-phosphate | 1.60 | 6.36 x 10^-3^ |
| D-Hexose-phosphate | 1.79 | 7.37 x 10^-3^ |
| Nicotinamide | -1.04 | 7.53 x 10^-3^ |
| 2-Keto-D-gluconate | -2.09 | 0.010 |
| 5'-methylthioadenosine | 1.94 | 0.011 |
| Glycerol-3-phosphate | 2.64 | 0.012 |
| Ornithine | 2.20 | 0.012 |
| Guanosine | 3.02 | 0.013 |
| Dihydroxyacetone phosphate (DHAP) | 1.89 | 0.015 |
| Pentose-phosphate | 2.02 | 0.018 |
| 2-Deoxyribose-1-phosphate | 1.43 | 0.019 |
| N-acetyl-glutamate | 1.59 | 0.020 |
| Citrate | 1.56 | 0.021 |
| Lysine | 3.16 | 0.022 |
| Glutamine | 2.65 | 0.023 |
| ADP | 3.92 | 0.028 |
| Glycerate-2,3-diphosphate | -1.06 | 0.032 |
| Stearate | -1.26 | 0.033 |
| UDP | 1.87 | 0.033 |
| GABA | 2.29 | 0.038 |
| Riboflavin (Vitamin B2) | 1.69 | 0.046 |
| Taurine | 2.37 | 0.048 |
| Adenosine | -1.44 | 0.049 |
| Inosine | 1.69 | 0.050 |
| Orotidine-phosphate | 1.12 | 0.050 |
| Palmitate | -1.08 | 0.053 |
| Aspartate | 1.64 | 0.062 |
| Reduced glutathione | 1.80 | 0.074 |
| Citraconate | -1.01 | 0.075 |
| D-Glucono-lactone-6-phosphate | 1.01 | 0.079 |
| TDP | -1.24 | 0.084 |
| DL-Acetylcarnitine | 2.30 | 0.084 |
| Asparagine | 1.67 | 0.085 |
| Ceramide | -1.14 | 0.085 |
| Carnitine | 1.48 | 0.089 |
| Glycerophosphocholine | 1.70 | 0.089 |
| Proline | 1.86 | 0.090 |
| dATP | 1.33 | 0.092 |
| Arginine | 1.67 | 0.101 |
| Nicotinate | 1.66 | 0.104 |
| Tyrosine | 1.74 | 0.105 |
| Glucose-6-phosphate | -1.02 | 0.108 |
| Choline | 1.63 | 0.128 |
| 5-methyl-tetrahydrofolate | -2.88 | 0.130 |
| dCDP | 1.21 | 0.131 |
| Pyruvate | 1.04 | 0.134 |
| Histidine | 1.64 | 0.153 |
| 4-Pyridoxate | 1.07 | 0.157 |
| Aconitate (*cis* and *trans*) | -1.29 | 0.193 |
| Citrulline | 1.00 | 0.198 |
| (Iso)Leucine | 1.40 | 0.199 |
| Glutamate | 1.59 | 0.205 |
| Pyridine-2,3-dicarboxylate | 1.00 | 0.206 |
| Acetyllysine | 1.67 | 0.223 |
| Pyridoxine | -1.18 | 0.224 |
| S-adenosyl-L-methionine (SAM) | 2.00 | 0.225 |
| Malate | 1.09 | 0.236 |
| Threonine | 2.37 | 0.260 |
| Glycerate-diphosphate (1,3 and 2,3) | 1.22 | 0.270 |
| N-acetyl-glutamine | 1.61 | 0.272 |
| Acetyl phosphate | 1.00 | 0.303 |
| Valine | 1.34 | 0.327 |
| Methionine | 1.59 | 0.339 |
| Homoserine | 1.59 | 0.340 |
| Phenylalanine | 1.41 | 0.343 |
| Cytidine | 1.74 | 0.373 |
| NAD | 1.07 | 0.381 |
| Fumarate, Maleate, and iso-Ketovalerate | -1.16 | 0.404 |
| 4-Hydroxybenzoate | -1.08 | 0.482 |
|  | | |
